# Leaving and staying with the employer—Changes in work, health, and work ability among older workers

**DOI:** 10.1007/s00420-020-01563-0

**Published:** 2020-09-06

**Authors:** Nina Garthe, Hans Martin Hasselhorn

**Affiliations:** Department of Occupational Health Science, School of Mechanical Engineering and Safety Engineering, University of Wuppertal, Gaußstraße 20, 42119 Wuppertal, Germany

**Keywords:** Employer change, Older workers, Job lock, Psychosocial work factors

## Abstract

**Objective:**

The aim of this prospective study was to examine employer changes among older workers and to relate them to psychosocial work factors, health, and work ability. Four groups of employees as elaborated by Hom et al. (2012) were distinguished: Enthusiastic leavers (EL), reluctant leavers (RL), enthusiastic stayers (ES), and reluctant stayers (RS).

**Methods:**

Repeated Measures ANOVA analyses were based on data from the second and third waves (2014, 2018) of the German lidA Cohort Study, a representative study of employees born in 1959 or 1965.

**Results:**

The largest proportion of participants was ES (73.3%), 13.2% stayed with their employer although they would have preferred to leave (RS). 7.1% changed employer between 2014 and 2018 voluntarily (EL), 6.4% involuntarily (RL). Analyses confirmed that the four groups already differed in 2014 in terms of health, work ability, and psychosocial work factors and that these outcomes change in different characteristic patterns over time. Most outcomes improved substantially following the change among EL. RS already reported poor outcomes in 2014 and exhibited a further deterioration while staying at the undesired workplace.

**Conclusion:**

Our findings indicate that an employer change is followed by improvements of work, health, and work ability. We conclude that an inclusive labor market policy for older workers allowing for high job mobility may have the potential to contribute to considerable improvements of workers’ individual working conditions, health, and work ability, thereby increasing the work participation. Also, the considerable group of RL requires increased political and scientific attention.

## Introduction

The demographic change in Germany leads to an aging and shrinking workforce. A consequence for many workers is the extension of their working lives. This raises the question on how older workers can manage to work until, or possibly longer than the statutory retirement age. According to the international JPI UEP working group “the positive impact of work factors that promote longer working lives and help retain workers should be given greater attention in retirement studies” (Hasselhorn and Apt 2015). One strategy proposed is the change of employer at higher working age. Such a change may exhibit the potential to improve the fit between the older workers and their work, with regard to work factors, qualifications, motivation, work ability, and health, and therefore to extend the personal working life (Behrens 1998; Jahn and Ulbricht 2011; Morschhäuser 2002).

When investigating employer changes among older workers, voluntary and involuntary changes need to be differentiated. While a voluntary change is often a planned transition, losing one’s job may often be unexpected, it can lead to unemployment, job search and–at best–to a new job with many uncertainties. This may constitute a substantial challenge–not least for older workers (Brauer and Clemens 2010)–and might rather bear health risks than benefits. However, comparative research on consequences of voluntary and involuntary changes is rare (Chadi and Hetschko 2014; Wagenaar et al. 2012).

But also voluntary employer changes offer not only chances but also bear risks, especially for older workers. Behrens (1998) pointed out that employer changes cannot be a general recommendation for all older workers who find themselves in inappropriate work situations. Beyond the risk of becoming unemployed, further obstacles keep older workers from changing, such as concerns about reduced pay after a change (Schneider 2010), the expectation of a temporary contract or a misfit of skills, and knowledge in the new job (Bailey and Hansson 1995). Morschhäuser (2006) described in her qualitative study that older workers with poor health and physically demanding work did not want to leave familiar workplaces and showed low confidence in managing a change. These psychological and further obstacles are covered by the theories on job lock and stuck at work, which point out that such a locked occupational situation may have negative impact on work and health (Huysse-Gaytandjieva et al. 2013). The aspect of involuntary staying with one’s employer in contrast to voluntary staying should thus also be considered when investigating employer changes and older workers’ work motivation, work ability, health, and employment perspective.

### Theoretical background and hypotheses

Based on their review of employee turnover, Hom et al. (2012) presented a theory on motivational states of staying and leaving, depicting four groups of employees with different cognitive states concerning staying with or leaving the employer. The combination of two dimensions, (a) desired staying or leaving and (b) high or low perceived control of this preference, leads to four groups covering the scenarios discussed above: Enthusiastic leavers (EL), who want to and can leave, reluctant leavers (RL), who have to leave because they are forced to, reluctant stayers (RS), who stay because they feel they cannot leave although they would prefer to, and enthusiastic stayers (ES), who want to stay and feel no external pressure to leave their employer.

In all groups, work factors, especially psychosocial work factors, as well as health and work ability play a central role. EL may want to leave their employer due to a lack of person–work fit and want improvements (Mobley 1977; Trevor 2001). The work situation is perceived similarly by RS, yet they cannot leave due to diverse obstacles. Workers belonging to this group may develop work avoidance and counterproductive workplace behaviors and quit psychologically (Mobley et al. 1979; Hulin et al. 1985; Mowday et al. 1982). In contrast, RL may have to leave their employer, for example, due to low performance, and have to find a new job, which may constitute a great challenge, not least for older workers (Jackofsky 1984; Bäcker et al. 2017). Finally, ES may have a satisfying person–work fit and good work performance (Mobley 1977; Lee et al. 1999).

Previous empirical studies usually examined singular groups of the four, primarily EL (Reineholm et al. 2012), the most frequent outcomes were mental health indicators (Liljegren and Ekberg 2008), and the most frequently investigated group are middle-aged employees (Rubenstein et al. 2018). Most studies are cross-sectional investigations using change proxies, such as job mobility intentions (Alcover and Topa 2018), instead of examining actual changes in longitudinal studies (Raeve et al. 2008).

This article aims to empirically investigate all four groups of EL, RS, RL, and ES in a longitudinal study in terms of differences and changes over time with respect to mental and physical health, work ability, and psychosocial work factors among older workers. The assumptions compiled by Hom et al. (2012) lead to two hypotheses:

H1: The groups differ significantly in terms of health, work ability, and psychosocial work factors.

H2: The groups change significantly differently over time in terms of health, work ability, and psychosocial work factors.

## Methods

### Data and sample

The analyses are based on data from the German lidA Cohort Study on Work, Age, Health and Work participation, a representative cohort study of older employees in Germany (www.lida-studie.de). The aim of lidA is to investigate work and employment in the aging workforce. Initially employed people subject to social security contributions (no self-employed or sworn civil servants), born in either 1959 or 1965, are interviewed every three to four years in their homes (computer-assisted personal interviewing, CAPI). The data used here are derived from the second and third waves of the study, 2014 (t1) and 2018 (t2) with 4244 and 3586 participants, respectively. In 2018 the participants were 53 and 59 years old. A more detailed description of the lidA Cohort Study and its design has been given elsewhere (Hasselhorn et al. 2014; Rauch et al. 2015).

In all, 3232 workers participated in t1 and t2. In order to focus on employer changes, study participants were excluded if they were not employed full time, part time, or marginally in any of the waves. As a result, the sample consists of 2811 participants.

## Measures

### Employer change groups

The change of employer was assessed in the third wave in 2018 (t2) by the question: “Have you changed your employer since the last interview? (Yes/No).” Participants, who reported a change, were asked whether they changed on their own initiative (enthusiastic leavers), or on the initiative of their employer (reluctant leavers). Participants, who reported no change, were asked whether they would have liked to change since the last study interview in 2014 (reluctant stayers) or not (enthusiastic stayers). Thus, the four groups differentiate the participants whether they changed or not and wanted to change or not between 2014 (t1) and 2018 (t2).

### Mental and physical health

The outcomes mental and physical health were assessed with the Short Form Health Survey (SF-12) (Nübling et al. 2006; Ware et al. 1995). Component scores ranging from 0 to 100 with a high score indicating better health were calculated. Both SF-12 scales were found to have acceptable psychometric properties and validity (Ware et al. 1996).

### Work ability

To measure work ability, the second dimension of the Work Ability Index was used, which consists of three questions. Two questions refer to the actual self-assessed work ability with respect to mental and physical demands at work, respectively. The answers were weighted by the response to a third question, indicating whether the participant is mainly mentally active in the main job, mainly physically active or both equally. The resulting sum score ranges from 2 (no work ability) to 10 (high work ability). The second dimension of the Work Ability Index was shown to be a suitable short measure for work ability in occupational health research and employee surveys (Ebener and Hasselhorn 2019).

### Psychosocial work factors

Psychosocial work factors were assessed with scales from the Copenhagen Psychosocial Questionnaire (COPSOQ-II, middle version, Pejtersen et al. 2010). Six psychosocial work factors were generated with scores ranging from 0 to 100: Leadership quality, social support from colleagues, work–family conflict, possibilities for development, quantitative demands, and influence at work. High scores indicate a high expression of the concept. A detailed description of the scale construction in lidA is given by Willner (2013). Following recommendations by Willner (2013), one item was deleted to generate the sum score for possibilities for development.

### Demographics and employment background information

Sociodemographic and employment background information from t1 was considered in the analyses. This includes gender (male/female), year of birth (1959/1965), vocational education (low: no qualification, vocational operational education; off-the-job training / medium: technical school; master school / high: higher vocational education, university education), and weekly working time (full time/part time/marginal employment). Additionally, seniority at t1, indicating the duration of employment with the same employer (quantified in years), was considered.

### Statistical analyses

First, sociodemographic and employment variables were tested for significant differences between the groups using the χ^2^ statistic and one-way analyses of variance.

Second, the group means of mental and physical health, work ability, and the six psychosocial work factors were compared across the three waves. GLM Repeated Measures ANOVAs were performed to investigate within group and between group differences occurring between t1 and t2. Three effects were tested: The main time effect, indicating a significant change of the outcome over time, the main group effect, indicating a significant difference between the four groups in the outcome, and the interaction effect group*time, indicating significant different group changes over time. In addition to the main group effect, Post hoc tests (Bonferroni corrected) were conducted to indicate which groups differ in which way from each other. A significant main group effect supports Hypothesis 1 and a significant interaction effect group*time supports Hypothesis 2. All statistical analyses were performed using SPSS version 25.0.

## Results

### Group descriptions

Of the eligible 2811 participants, 13.5% changed employer between t1 and t2, 7.1% were EL and 6.4% were RL (Table 1). The largest proportion of participants was ES (73.3%), 13.2% stayed with their employer although they preferred to leave (RS). Among EL there were more women and among ES more older participants than in the other groups. Participants with low vocational education were overrepresented in RS and marginal workers in EL. Seniority at t1 was in both leavers’ groups, EL and RL, substantially lower than in RS and ES.

**Table 1:** Sample and group characteristics

	Sample (*n* = 2811, 100.0%)		Enthusiastic leavers (*n* = 199, 7.1%)		Reluctant leavers (*n* = 179, 6.4%)		Reluctant stayers (*n* = 370, 13.2%)		Enthusiastic stayers (*n* = 2063, 73.4%)	
	%	M (SD)	%	M (SD)	%	M (SD)	%	M (SD)	%	M (SD)
Gender* ^a^										
Male	44.9		34.7		43.0		43.0		46.4	
Female	55.1		65.3		57.0		57.0		53.6	
Year of birth***										
1959	44.9		35.2		34.1		38.4		47.9	
1965	55.1		64.8		65.9		61.6		52.1	
Vocational education										
Low	20.3		19.8		27.5		21.3		19.6	
Medium	56.8		58.9		51.1		57.9		56.9	
High	22.9		21.3		21.3		20.8		23.6	
Weekly working time***										
Full time	66.7		54.8		66.5		68.1		67.6	
Part time	29.0		35.2		26.3		30.3		28.5	
Marginal employment	4.3		10.1		7.3		1.6		3.9	
Seniority***		16.3 (10.4)		8.8 (8.0)		10.1 (9.3)		15.1 (9.4)		17.8 (10.4)

*M* mean, *SD* standard deviation

^*a*^Chi-square or one-way ANOVA significant group difference, *p < .05, ***p < .001

#### H1. Group differences in health, work ability, and psychosocial work factors

Sample and group means as well as confidence intervals for mental and physical health, work ability, and the six psychosocial work factors at t1 and t2 are shown in Table 2. The results of the Repeated Measures ANOVA support H1 (main group effect, Table 3): The groups differ significantly in terms of health, work ability, and psychosocial work factors. Notably, the main group effect of leadership quality shows a high effect size (η^2^ = 0.08) in contrast to the other outcomes. The Post hoc tests indicate that in all cases the group of RS significantly differ from one or more other groups. This group exhibited the most adverse mean scores for work ability, the health indicators, and all psychosocial work factors.

**Table 2:** Group means and confidence intervals

		Sample		Enthusiastic leavers (EL)			Reluctant leavers (RL)		Reluctant stayers (RS)		Enthusiastic stayers (ES)	
			95% CI		95% CI			95% CI		95% CI		95% CI
Mental health	*n*	2798		196			169		364		2045	
	*t1*	50.6	48.6– 52.5	49.0	47.4– 50.6		50.1	48.5– 51.8	48.3	47.2– 49.4	51.2	50.8– 51.6
	*t2*	51.7	49.8– 53.6	53.7	52.4– 55.0		50.6	49.1– 52.1	48.4	47.4– 49.5	52.1	51.7– 52.5
Physical health	*n*	2798		196			169		364		2045	
	*t1*	49.9	48.2– 51.7	51.9	50.7– 53.0		48.8	47.3– 50.2	49.2	48.3– 50.1	50.0	49.6– 50.3
	*t2*	48.1	46.3– 49.9	49.2	47.9– 50.4		47.6	46.2– 49.0	47.0	46.0– 47.9	48.3	47.9– 48.7
Work ability	*n*	2803		196			171		363		2044	
	*t1*	8.0	7.7– 8.2	7.9	7.7– 8.2		7.7	7.4– 8.0	7.6	7.4– 7.8	8.0	8.0–8.1
	*t2*	7.8	7.5– 8.1	8.3	8.1– 8.5		8.0	7.7– 8.3	7.2	7.0–7.4	7.8	7.7– 7.9
Leadership quality	*n*	2748		187			161		360		2011	
	*t1*	54.4	49.8– 58.9	50.9	47.5– 54.3		50.3	46.3– 54.3	43.4	40.9– 45.9	57.1	56.1– 58.0
	*t2*	53.0	48.3– 57.5	59.4	55.8– 63.0		56.1	52.5– 59.7	36.5	34.3– 38.9	55.1	54.1– 56.0
Support from colleagues	*n*	2694		179			156		351		1957	
	*t1*	72.6	68.5– 77.2	73.5	70.1– 76.9		72.8	69.1– 76.4	68.8	66.3– 71.4	73.2	72.2– 74.1
	*t2*	76.1	72.2– 80.3	79.8	76.9– 82.7		75.8	72.3– 79.4	69.9	67.5– 72.3	76.9	76.0– 77.8
Work-family conflict	*n*	2804		196			178		369		2059	
	*t1*	35.0	29.7– 40.3	36.7	32.9– 40.6		34.9	30.5– 39.3	40.7	38.0– 43.4	33.8	32.7– 34.9
	*t2*	36.5	31.4– 41.9	30.8	27.2– 34.5		35.9	31.8– 40.0	46.8	44.1– 49.5	35.4	34.3– 36.6
Possibilities for development	*n*	2809		196			171		364		2049	
	*t1*	63.1	59.0– 67.2	58.5	55.1– 61.9		60.8	57.3– 64.2	60.9	58.7– 63.1	64.1	63.2– 64.9
	*t2*	62.7	58.9– 66.5	65.7	62.8– 68.6		62.3	59.1– 65.5	55.8	53.8– 57.8	63.7	62.9– 64.5
Quantitative demands	*n*	2808		197			171		364		2051	
	*t1*	45.7	41.1–50.2	46.3	42.9– 49.6		39.4	35.8– 43.2	49.6	47.2– 51.9	45.4	44.5– 46.4
	*t2*	45.3	41.0– 49.5	39.7	36.8– 42.7		39.6	36.1– 43.2	51.4	49.3– 53.6	45.2	44.3– 46.1
Influence at work	*n*	2809		196			171		364		2050	
	*t1*	38.8	33.7–44.0	38.4	34.5– 42.2		35.5	31.9– 39.2	36.5	33.9– 39.0	39.6	38.5– 40.8
	*t2*	36.2	31.2– 41.2	37.1	33.3– 41.0		32.7	29.1– 36.4	30.9	28.5– 33.2	37.4	36.3– 38.5

**Table 3:** Repeated Measures ANOVA

		Hypothesis supported	Significant post-hoc comparison at *p* > .05 (Bonferroni-corrected)
*Mental health*			
Main time effect	F(1, 2787) = 27.19, *p* < .001, partial η^2^ = .01	–	–
Main group effect	F(3, 2787) = 16.36, *p* < .001, partial η^2^ = .02	Yes	ES > RS, EL > RS
Interaction effect group*time	F(3, 2787) = 9.54, *p* < .001, partial η^2^ = .01	Yes	–
*Physical health*			
Main time effect	F(1, 2787) = 59.11, *p* < .001, partial η^2^ = .02	–	–
Main group effect	F(3, 2787) = 4.98, *p* < .01, partial η^2^ = .01	Yes	EL > RS, EL > RL
Interaction effect group*time	F(3, 2787) = 1.42, *p* = .236, partial η^2^ = .00	No	–
*Work ability*			
Main time effect	F(1, 2793) = 1.75, *p* = .186, partial η^2^ = .00	–	–
Main group effect	F(3, 2793) = 20.09, *p* < .001, partial η^2^ = .02	Yes	ES > RS, EL > RS, RL > RS
Interaction effect group*time	F(3, 2793) = 12.22, *p* < .001, partial η^2^ = .01	Yes	–
*Leadership quality*			
Main time effect	F(1, 2698) = 4.57, *p* < .05, partial η^2^ = .00	–	–
Main group effect	F(3, 2698) = 73.73, *p* < .001, partial η^2^ = .08	Yes	ES > RS, EL > RS, RL > RS
Interaction effect group*time	F(3, 2698) = 23.42, *p* < .001, partial η^2^ = .03	Yes	–
*Support from colleagues*			
Main time effect	F(1, 2628) = 19.65, *p* < .001, partial η^2^ = .01	–	–
Main group effect	F(3, 2628) = 10.32, *p* < .001, partial η^2^ = .01	Yes	ES > RS, EL > RS, RL > RS
Interaction effect group*time	F(3, 2628) = 1.87, *p* = .133, partial η^2^ = .00	No	–
*Work-family conflict*			
Main time effect	F(1, 2795) = 0.79, *p* = .374, partial η^2^ = .00	–	–
Main group effect	F(3, 2795) = 17.26, *p* < .001, partial η^2^ = .02	Yes	ES < RS, EL < RS, RL < RS
Interaction effect group*time	F(3, 2795) = 8.49, *p* < .001, partial η^2^ = .01	Yes	––
*Possibilities for development*			
Main time effect	F(1, 2805) = 2.20, *p* = .138, partial η^2^ = .00	–	–
Main group effect	F(3, 2805) = 10.97, *p* < .001, partial η^2^ = .01	Yes	ES > RS
Interaction effect group*time	F(3, 2805) = 18.95, *p* < .001, partial η^2^ = .02	Yes	–
*Quantitative demands*			
Main time effect	F(1, 2804) = 3.68, *p* = .055, partial η^2^ = .00	–	–
Main group effect	F(3, 2804) = 14.68, *p* < .001, partial η^2^ = .02	Yes	ES < RS, ES > RL, EL < RS, RL < RS
Interaction effect group*time	F(3, 2804) = 7.24, *p* < .001, partial η^2^ = .01	Yes	–
*Influence at work*			
Main time effect	F(1, 2803) = 17.32, *p* < .001, partial η^2^ = .01	–	–
Main group effect	F(3, 2803) = 6.09, *p* < .001, partial η^2^ = .01	Yes	ES > RS
Interaction effect group*time	F(3, 2803) = 2.25, *p* = .081, partial η^2^ = .00	No	–

*EL* enthusiastic leavers, *RL* reluctant leavers, *ES* enthusiastic stayers, *RS* reluctant stayers

#### H2. Group changes over time

The Repeated Measures ANOVAs support H2 for most outcomes (interaction effect group*time, Table 3): The groups differ significantly with respect to changes over time in terms of mental, but not physical health, in terms of work ability and the psychosocial work factors, leadership quality, work–family conflict, possibilities for development, and quantitative demands. Again, the greatest effect size was found for the interaction effect for leadership quality (η^2^ = 0.03).

Table 2 provides insight of these changes: In the group of EL, the ratings for the new job at t2 indicate substantial improvements for mental health, work ability, and leadership quality, work–family conflict, possibilities for development, and quantitative demands in relation to the previous job (t1). RL reported, on one hand, improvements in work ability, leadership quality, and support from colleagues, and on the other hand, deteriorations in influence at work. RS reported deteriorations in leadership quality, possibilities for development, influence at work, and work–family conflict. Among ES the mean scores for the outcomes changed only slightly over time.

## Discussion

In our analyses we find that the four employer change groups depicted by Hom et al. (2012) already differ at t1 in terms of health, work ability, and psychosocial work factors and that these outcomes change in different characteristic patterns over time. There were only marginal changes of outcomes in the group of ES. Most outcomes improved substantially over time among EL, some also among RL. RS already reported poor outcomes in 2014 and exhibited a further deterioration while staying at the undesired workplace.

In relation to economically liberal countries, the frequency of employer changes tends to be low in the German labor market (Buchholz 2008). However, in our study, the proportion of EL (7.1%) and RL (6.4%) over four years is notably high, considering that older employees, in particular, were found to have substantial obstacles to employer change and change rarely (Bailey and Hansson 1995; Carless and Arnup 2011). The high proportion of RS found in the study (13.2%), however, may be interpreted as the effect of the obstacles mentioned above.

### Enthusiastic leavers

As theorized by Hom et al. (2012), the psychosocial work factors improved substantially with a voluntary change of employer. At t1, several factors clearly showed more adverse mean scores among the EL than for RS or ES, namely leadership quality, possibilities for development, and work–family conflict, all established causes for voluntary change (Raeve et al. 2008; Rubenstein et al. 2018; Nouri and Parker 2013). Also work ability and mental health improved strongly after the change, but not physical health, indicating the relevance of differentiation of health when investigating work and health. These results are in line with those from Liljegren and Ekberg (2008) who found job mobility to be a predictor of mental, but not physical health, though mental health not as a predictor of job mobility.

Another observation makes the group of EL outstanding: At t2 the EL reported the best mental health, work ability and leadership quality, and the lowest work–family conflict of all four groups depicted by Hom et al. (2012).

### Reluctant leavers

According to our findings, the group of RL is, before the change, characterized by low leadership quality, low influence at work, and very low quantitative demands. However, the involuntary change seems to go along with considerable improvements, such as work ability, leadership quality, and support from colleagues, but also deteriorations for influence at work. Our analyses can neither confirm nor reject the assumptions that RL were low performers as described in Jackofsky´s model on turnover and job performance from 1984.

There were no changes in the two health outcomes over time, showing the importance of a conceptual distinction of health and work ability. Even if the workers’ health does not change, work ability can be improved by adapting the work situation (Ebener and Hasselhorn 2016).

### Reluctant stayers

Already at t1, the group of RS stands out with respect to several work exposures and outcomes and stands particularly in contrast to the EL. In terms of leadership quality, work–family conflict, possibilities for development, quantitative demands, and influence at work, this group already exhibited poor or even worst mean scores in the sample which then further deteriorated over the next four years. These results may reflect lack of person–work fit in this group as concluded by Hom et al. (2012) and also assumptions based on the job lock and stuck at work theories (Huysse-Gaytandjieva et al. 2013): Specifically those with poor work find it difficult to change to a better job due to a lack of opportunities and low qualifications.

Also work ability declined in this group which is in line with conclusions from the international JPI UEP working group, that work ability declines with age, especially in jobs with physically strenuous tasks and that some older workers may be “locked” in such jobs (Hasselhorn and Apt 2015). In terms of health, our results indicate stable mental health over time, at a very low level, however, and a deterioration of physical health, which is of comparable size as in the other groups. A Swedish working group found that being locked-in is detrimental to well-being (Stengård et al. 2016).

### Enthusiastic stayers

As described by Hom et al. (2012) the group of ES differs clearly from all others: While there are many significant and different changes over time among EL, RL, and RS, only very small changes were found in this largest group, the ES. Although it is a large group, deteriorations in physical health and work ability were found, which may be attributed to aging (Kooij 2015). Notable is that the ES had the highest seniority at t1 (Table 1), which may be indicative of a long-lasting person–work fit for many (Hom et al. 2012).

## Strengths and limitations

The strength of the lidA study is that the four occupational change groups suggested by Hom et al. (2012) can be identified and examined in depth, over time and among older workers, because of the large age-homogeneous sample size and the longitudinal study design. Another advantage is the representativeness of the sample for the older German socially insured working population of similar age. Limitations are that the study focuses on psychosocial work factors only, did not include employees, who became unemployed, and that the different group sizes may impede comparability between the groups of EL, RL, and RS and the greatest group of ES.

## Conclusions

Concluding, we confirm theoretical suggestions that a change of employer may lead to considerable improvements among a range of psychosocial work factors for older workers, especially when the step is taken voluntarily, but also following reluctant leaving. Our research results imply that older workers generally take the initiative to change their employer because they want to improve adverse psychosocial working conditions. Yet, voluntary changes have the potential to improve mental health and work ability as well.

If both changing groups–those changing voluntarily and involuntarily–benefit from an employer change, we may conclude that an inclusive labor market policy for older workers allowing for high job mobility may have the potential to contribute to considerable improvements of workers’ individual working conditions, health, and work ability, thereby increasing work participation of older workers and extending working lives.

Further, our results indicate that the group of reluctant stayers requires special attention from employers and policy and might also benefit from an inclusive labor market policy. This group of workers rates its own work situation increasingly poorer while staying at the undesired workplace. It may pose a risk group with regard to work ability, work motivation, and therefore employment participation at higher working age.

Considering the overall relevance of this topic and the growing availability of good data, research should dedicate more resources to this field. Thereby, research should differentiate voluntary and involuntary changes and not overlook the great risk group of reluctant stayers. Conceptually, physical and mental health should be differentiated from work ability. Finally, besides investigating the determinants, future studies should look into the short- and long-term consequences of actual and desired employer changes among older workers and their relation to working life duration and quality.
